# Direct evaluation of MR-derived attenuation correction maps for PET/MR of the mouse myocardium

**DOI:** 10.1186/2197-7364-1-S1-A85

**Published:** 2014-07-29

**Authors:** Eleanor Evans, Guiso Buonincontri, Rob C Hawkes, Richard E Ansorge, T Adrian Carpenter, Stephen J Sawiak

**Affiliations:** Wolfson Brain Imaging Centre, University of Cambridge, Cambridge, UK; Department of Physics, University of Cambridge, Cambridge, UK; Behavioural and Clinical Neurosciences Institute, University of Cambridge, Cambridge, UK

Attenuation correction (AC) must be applied to provide accurate measurements of PET tracer activity concentrations. Due to the limited space available in PET/MR scanners, MR-derived AC (MRAC) is used as a substitute for gold standard transmission source scans [1]. We compared MRAC to transmission scans to evaluate its performance in mouse myocardium studies.

PET SUV values derived for 10 mice [2] using whole body MRAC maps were compared to those attained using AC maps from a transmission source. 3D FISP was acquired using a 4.7T Bruker BioSpec before the mouse was transferred on a standard Bruker animal bed (with single loop surface coil) to the Cambridge split magnet PET/MR [3]. A 10 minute transmission scan (^68^Ge) was performed. Emission data was acquired for 45 minutes following ~25MBq ^18^F-FDG administration.

*MRAC comparison* Following co-registration using SPMMouse [4], MR data were forward projected into 3D PET sinograms and thresholded to create an AC map, defined as a single region of tissue with uniform attenuation co-efficient of 0.095cm^–1^. SUV values were calculated from summed PET images (last 20 minutes) and compared on a voxel by voxel basis between images without AC, with transmission source AC, and with MRAC.

A 22.6 ± 0.9% (mean ± SD) improvement in mouse myocardium SUV values (shown in Figures 1 and 2) was seen by applying transmission AC and a 18.5 ± 0.9% improvement using MRAC, compared to not applying AC. The global attenuation correction over the whole mouse body was 20.7 ± 0.7% using transmission AC and 16.5 ± 1.3% using MRAC. Differences of up to 40% (mean: 30.1 ± 4.4%, range: 27-40%) were seen adjacent to the RF coil (see Figure 3).

**Figure 1:**
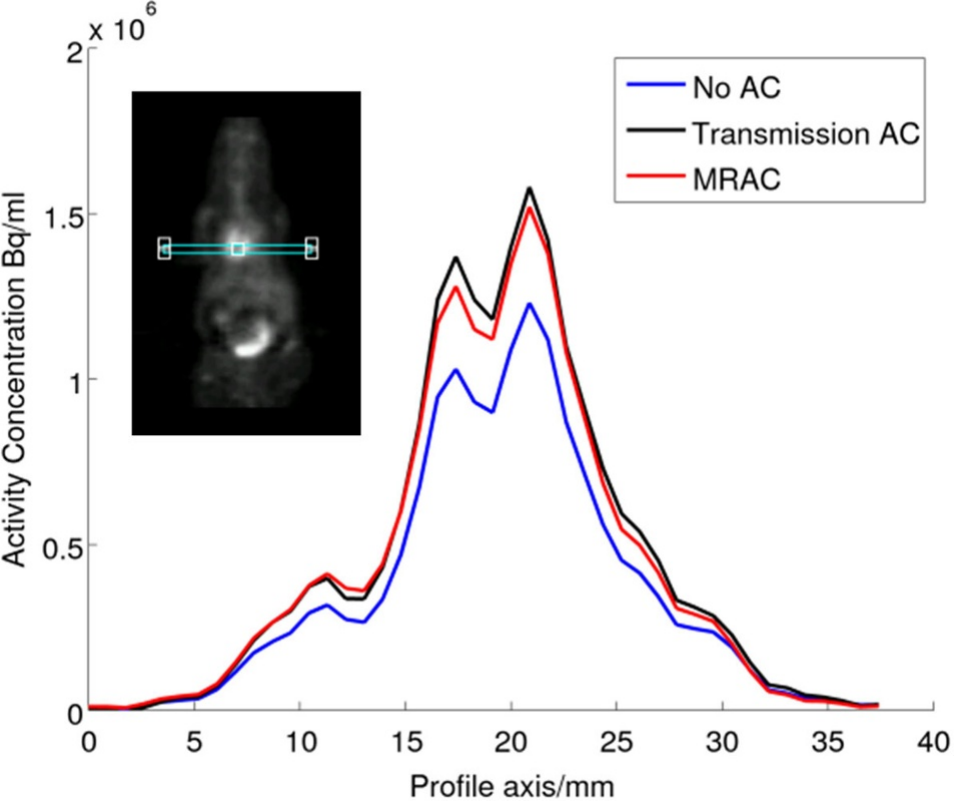
Single subject line profile for each AC method.

**Figure 2:**
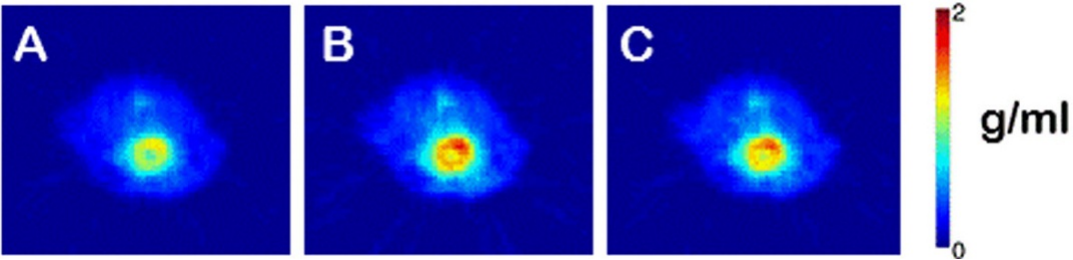
SUV maps (transverse view) for single subject. (A) No AC applied, (B) Transmission AC, (C) MRAC.

**Figure 3:**
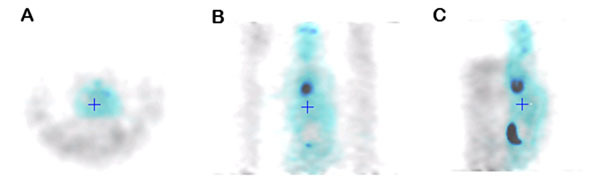
PET emission (blue) and transmission (grey) fused images showing mouse, coil and bed attenuation, (A) Transverse, (B) Coronal, (C) Sagittal.

## Conclusion

A simple, one region MRAC approach provided acceptable AC compared to transmission scanning for myocardial imaging in mice.
